# Reliability-aware fusion of ocular thermal imaging and radiometric features for proof-of-concept classification of diabetic retinopathy vs. healthy controls

**DOI:** 10.3389/fmed.2026.1888280

**Published:** 2026-08-10

**Authors:** Persiya J, Sasithradevi A, Anand Rajendran

**Affiliations:** 1 School of Electronics Engineering, Vellore Institute of Technology, Chennai, Tamil Nadu, India; 2 Centre for Advanced Data Science, Vellore Institute of Technology, Chennai, Tamil Nadu, India; 3 Department of Vitreo-Retina Services, Aravind Eye Hospital, Chennai, Tamil Nadu, India

**Keywords:** diabetic retinopathy affected eye, multimodal deep learning, ocular thermography, radiometric temperature features, thermal imaging

## Abstract

**Introduction:**

Early detection of diabetic retinopathy (DR) is crucial for preventing permanent vision impairment, yet widely used screening techniques such as fundus photography and optical coherence tomography (OCT) depend on expensive equipment and expert clinical interpretation, restricting their use in large-scale and low-resource environments. To overcome these challenges, this study introduces ReliaFusion-Net, a reliability-aware multimodal framework for proof-of-concept binary classification of eyes with DR vs. healthy controls, using thermal eye images and radiometric temperature features.

**Methods:**

The proposed ReliaFusion-Net architecture combines bidirectional co-attention with feature-wise linear modulation to effectively model complementary relationships between thermal image features and physiological temperature descriptors. A clinically curated dataset containing 558 thermal eye images, including 278 normal and 280 diabetic samples, was collected with ophthalmologist support. The framework was trained and evaluated for binary classification, and cross-validation experiments were conducted to assess model generalization.

**Results:**

Using an EdgeNeXt backbone, the proposed model achieved 93.18% accuracy, 93.26% F1-score, and 0.9785 AUC on the test set. Cross-validation results further confirmed strong generalization performance. These findings indicate that the reliability-aware multimodal fusion strategy effectively discriminates eyes with DR from healthy controls using non-invasive thermal imaging data.

**Discussion:**

By dynamically adapting modality contributions based on reliability, ReliaFusion-Net overcomes limitations associated with traditional fixed fusion strategies and improves classification robustness. Overall, the findings demonstrate proof-of-concept of reliability-aware multimodal learning to distinguish eyes with DR from healthy controls using non-invasive thermal imaging, with potential applicability in tele-ophthalmology settings. Future work focuses on multi-stage disease classification and longitudinal disease progression analysis.

## 1 Introduction

Diabetic retinopathy (DR) remains one of the major causes of preventable vision loss among working-age adults, and its prevalence continues to grow alongside the global rise in diabetes (1). A large-scale meta-analysis reported that roughly 103 million adults were affected by DR in 2020, with the number expected to increase in parallel with the diabetes epidemic (2). According to the International Diabetes Federation (IDF) Diabetes Atlas (11th edition, 2025), an estimated 11.1% of adults aged 20–79 years—about 589 million people—are currently living with diabetes, and more than 40% of these cases are undiagnosed. The same report projects that the global diabetic population may reach nearly 853 million by 2050 (3). These trends highlight the urgent need for scalable and reliable screening programs. Clinical guidelines further emphasize that timely detection, when patients often show no symptoms, can prevent sight-threatening complications (4). Pathophysiologically, DR is characterized by progressive microvascular injury initiated by chronic hyperglycemia, including pericyte loss, capillary basement membrane thickening, endothelial dysfunction, and capillary nonperfusion, leading to breakdown of the blood-retinal barrier (5–7). The resulting retinal ischemia triggers inflammatory cascades and pathological neovascularization, manifesting clinically as diabetic macular edema (DME) and proliferative DR (PDR). These microvascular and inflammatory changes alter local tissue perfusion and metabolic activity, creating hemodynamic perturbations that may produce detectable thermal signatures at the ocular surface providing a rationale for thermal imaging as a non-invasive functional biomarker (8, 9).

Conventional DR screening relies on retinal color fundus photography with human grading or automated analysis, whereas advanced structural imaging modalities, such as optical coherence tomography (OCT) and OCT angiography (OCTA), provide quantitative biomarkers, including central retinal thickness, vessel density, and foveal avascular zone metrics, that track disease progression (10). However, uptake of these screening approaches remains uneven globally due to infrastructure, workforce, and cost constraints (11). Emerging AI-driven technologies and teleophthalmology are expanding accessibility (12, 13), yet infrastructure and cost barriers persist in many settings. Infrared ocular thermography (IRT) offers a complementary, non-contact functional modality that maps ocular surface temperature (OST)—a physiologic surrogate reflecting ocular blood flow, autonomic regulation, and inflammatory status (14–16). While thermography alone has modest standalone screening utility, its sensitivity to hemodynamic and inflammatory perturbations makes it a promising functional surrogate biomarker. Notably, OST measurements are protocol-sensitive (ambient temperature, humidity, emissivity, calibration), necessitating robust, reliability-aware algorithms when deployed in screening pipelines (17). In pilot and clinical studies, eyes with DR exhibit consistently lower OST than controls and altered thermal responses to physiological or pharmacologic stimuli, consistent with impaired microvascular hemodynamics (18, 19). The control data also demonstrate systemic thermographic differences in people with type 2 diabetes at periocular and peripheral sites. It is important to note that ocular thermography measures surface temperature and does not directly image retinal vasculature. The rationale for its use in diabetic retinopathy screening is based on indirect physiological manifestations, including diabetes-related autonomic dysfunction, altered ocular blood flow, inflammatory responses, and impaired heat dissipation, which collectively influence ocular surface thermal patterns (6, 7). Accordingly, thermography is considered a functional screening adjunct rather than a direct modality for retinal lesion detection (5). The modest standalone discriminative power of thermal features urges the need for multimodal approaches that fuse thermal imaging with temperature spatial information to achieve clinically viable screening performance.

Early thermography studies established that eyes with DR exhibit lower OST across corneal and conjunctival regions and a blunted rise in temperature after pharmacological dilation, changes attributed to impaired retinal perfusion and autonomic regulation (20, 21). Sodi et al. and subsequent confirmatory work demonstrated consistent temperature depression in DR eyes, laying the physiological groundwork for automated thermal image analysis (18). Building on this foundation, many researchers have explored feature-engineered machine learning approaches using ocular thermal images for DR screening. Rajagopalan et al. reported one of the first end-to-end screening pipelines based on real-time thermal eye images acquired from 20 DR patients and 16 controls (22). After careful pre-processing and data augmentation, three classes of texture descriptors—gray level co-occurrence matrix (GLCM), Scale-invariant feature transform (SIFT), and Gabor filters—were extracted and classified with support vector machines (SVMs) using multiple kernels. Among these, Gabor features with a linear SVM achieved the best performance (≈95.7% accuracy, 98% sensitivity, and 93% specificity), followed by SIFT (~93% accuracy), while GLCM lagged (~72% accuracy with an RBF kernel). This study demonstrated the feasibility of binary DR vs. normal eye classification from thermal images, but it relied on a small, single-center dataset (72 original images) and operated only at the disease/no-disease level, limiting generalizability. To capture subtler disease signatures, Meenakshi et al. introduced a bispectrum-based higher-order spectral analysis framework (23). Using 125 thermal images evenly distributed among healthy eyes, DR, DME, dry age-related macular degeneration (AMD), and wet AMD, they applied Radon transformation followed by direct, indirect, and ARMA bispectrum computations and kernel principal component analysis. Decision-tree and k-nearest-neighbor classifiers trained on these features achieved up to 100% sensitivity, specificity, and accuracy for DR, AMD, and wet AMD, and 93% accuracy for DME. By exploiting third-order statistics that encode nonlinear frequency interactions, this work showed that bispectral features can effectively separate multiple retinal vascular diseases from thermal imagery. Nevertheless, the modest sample size and lack of external validation temper conclusions about real-world deployment. Sinha et al. (24) complementary large-scale evidence comes from a multicenter Indian case–control study of 1,095 adults, which measured skin temperature at ocular and peripheral sites using infrared thermography. Diabetic subjects exhibited significantly lower mean temperatures at the medial canthus, forehead, and other regions, with weak but significant negative correlations to glycated hemoglobin (HbA1c). However, receiver operating characteristic analysis yielded only modest diagnostic power (area under the curve 0.54–0.58). Taken together, these studies demonstrate a clear evolution in the field. Table 1 summarizes the previous studies on diabetic ocular conditions using thermal images.

**Table 1:** Summary of existing thermal imaging studies on diabetic ocular conditions, highlighting datasets, methods, key findings, and limitations.

References	Disease study	Dataset detail	Method	Inference	Limitation
Madura Meenakshi et al. (23)	Diabetic Retinopathy, Diabetic Macular Edema, Age-Related Macular Degeneration (AMD - dry & wet)	Normal – 25 DR – 25 DME – 25 AMD Dry – 25 AMD Wet – 25 Total – 125 Images	Feature-based machine learning	Bispectrum-based spectral analysis captured non-linear thermal patterns for accurate retinal disease detection, though evaluation was limited to a binary classification setup.	Small dataset (125 images) limits generalizability.
Madura Meenakshi et al. (19)	Diabetic Retinopathy	Normal – 64 images, diabetes mellitus without DR – 60, diabetes mellitus with DR – 36 Total – 160 images	Statistical/Feature analysis	Quantitative thermography of corneal and lacrimal sac regions non-invasively detects early physiological changes associated with diabetic retinopathy.	Limited sample size and no staging of DR severity.
Sinha et al. (24)	Type 2 Diabetes Mellitus	Healthy controls – 505, Type 2 diabetes people – 590.	Feature based Statistical analysis	Diabetic subjects show significantly lower skin temperatures at forehead, medial canthus, pinna, and palmar hand surfaces, with negative correlations to HbA1C.	Consensus-based site selection, Weak correlation.
Rajagopalan et al. (22)	Diabetic Retinopathy	Control – 32, DR – 40 Total – 72 images	Feature-based machine learning	Combining infrared thermal imaging with Gabor texture features and SVM offers a non-invasive approach for diabetic retinopathy screening.	Limited dataset size, no external validation; limited feature-engineering scope
Naidorf-Rosenblatt et al. (6)	AMD and DR	Control – 56, DR – 97, AMD – 163, Total – 315 images	OST-based statistical analysis	OST comparison across DR, AMD, and healthy groups showed lower temperatures in DR eyes, while those with macular edema exhibited higher OST than DR without edema.	Limited dataset size,
Chandrasekar et al. (20)	Diabetic Retinopathy (NPDR & PDR)	Normal participants - 80 NPDR - 50 PDR – 20 Total – 150 images	Statistical analysis of OST	DR eyes exhibit lower ocular surface temperature and reduced thermal responsiveness, reflecting altered ocular and systemic vascular regulation and supporting OST as a complementary, non-invasive indicator in DR screening.	Small dataset, especially the PDR samples were less, limits generalizability.
Sodi et al. (18)	NPDR	Healthy – 53 NPDR – 51 Total – 104 images	Statistical analysis	Infrared thermography showed consistently lower ocular surface temperatures in diabetic eyes compared to controls across all measured regions.	Limited Dataset size

Previous thermography-based diabetic screening retinopathy classification studies were primarily constrained by limited dataset size and conventional feature-engineering methods. DR, diabetic retinopathy; DME, diabetic macular edema; AMD, age-related macular degeneration; NPDR, non-proliferative diabetic retinopathy; PDR, proliferative diabetic retinopathy; OST, ocular surface temperature.

Recent advances in fundus-image-based diabetic retinopathy diagnosis have focused on uncertainty-aware learning, explainable AI, graph-based deep learning, and transformer-based architectures to enhance diagnostic reliability and interpretability. These developments emphasize the importance of trustworthy and clinically explainable ophthalmic AI systems (25, 26). Early investigations revealed distinct thermal changes linked to diabetic eye. Later studies using methods such as texture-based SVMs and bispectrum-driven higher-order spectral analysis with decision trees and k-nearest neighbors achieved good accuracy on small, controlled datasets and even distinguished multiple ocular diseases. However, major challenges remain: the scarcity of large-scale datasets with robust clinical labeling, strong dependence on environmental factors like temperature and humidity. Moreover, no multimodal analyses combining thermal and complementary modalities have been conducted, and there are currently no publicly available thermal eye image datasets specifically curated for normal vs. eye with DR classification, limiting reproducibility.

To address these challenges, the study introduces ReliaFusion-Net, a multimodal deep learning framework that fuses thermal eye images with temperature-related features. ReliaFusion-Net learns sample-specific modality reliability scores, aligns them with predictive importance, and applies bidirectional co-attention with Feature-wise Linear Modulation (FiLM) to integrate temperature into image features. A clinically curated dataset comprising 558 thermal eye images (278 Normal and 280 diabetic) was developed in collaboration with ophthalmologists. This dataset was used for binary classification of eyes with DR versus healthy eyes, establishing a proof-of-concept foundation for thermography-based classification method. The major contributions of the current work are:

- Clinically validated self-curated thermal image dataset for normal vs. eye with DR classification.
- Multimodal analyses combining thermal image and radiometric temperature data.
- A deep learning-based ReliaFusion-Net framework incorporating harmonic co-attention and modality reliability for robust classification.
- Detailed performance analysis with cross-validation.

## 2 Methodology

**Figure 1:**
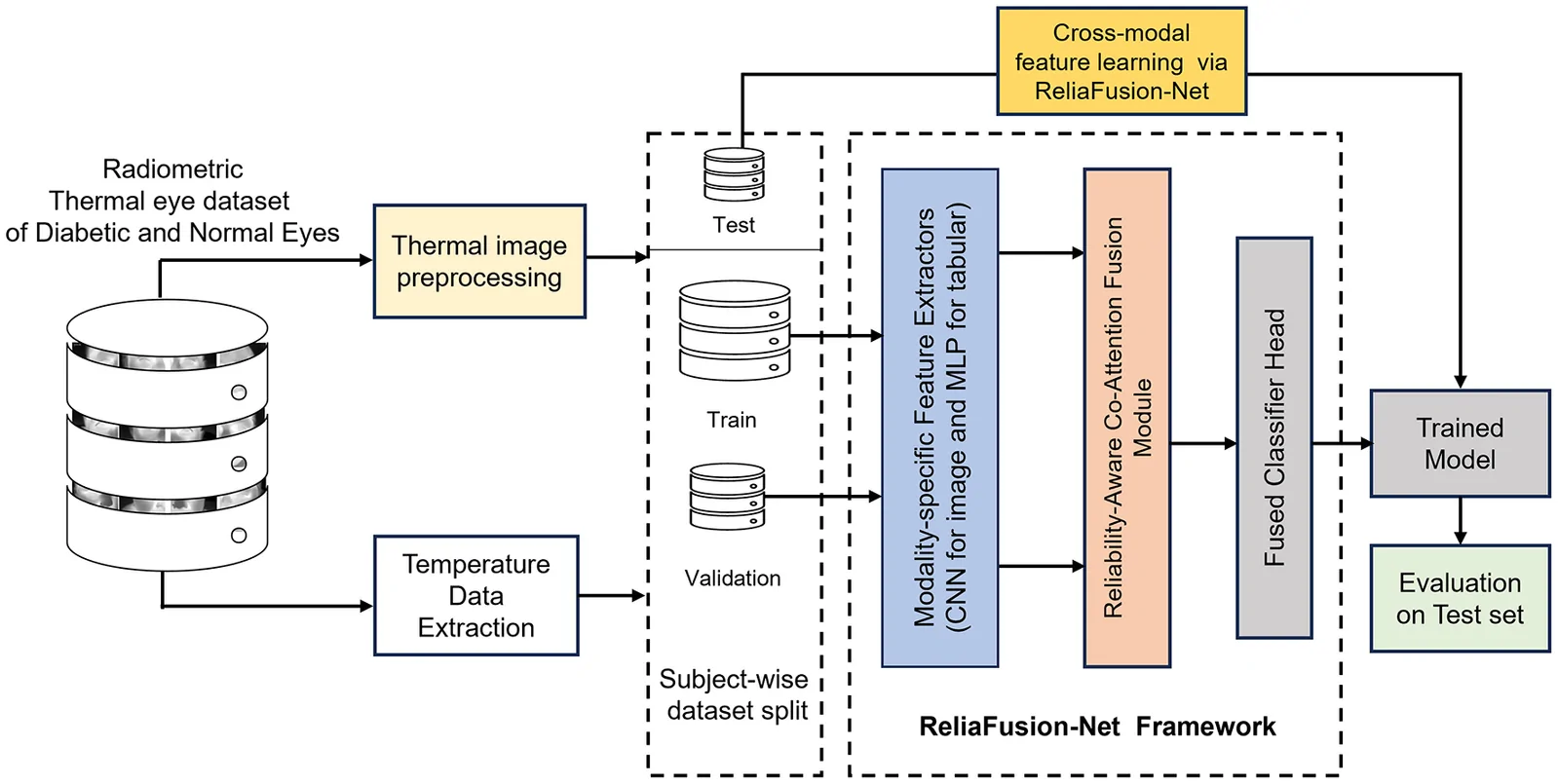
Overall flow diagram of the proposed ReliaFusion-Net framework.

The proposed framework integrates thermal imaging and temperature data for binary DR-vs.-normal classification. As illustrated in Figure 1, thermal eye images undergo preprocessing and subject-wise dataset splitting into training, validation, and testing sets. Modality-specific feature extractors—Convolutional Neural Networks (CNNs) for thermal images and a multilayer perceptron (MLP) for tabular temperature data features are employed. These representations are combined through a reliability-aware co-attention fusion module that captures inter-modality dependencies and mitigates noise from individual sources. The fused features are subsequently passed through a classification head to produce the final prediction, which is evaluated on the independent test set.

### 2.1 Data curation process and pre-processing

**Figure 2:**
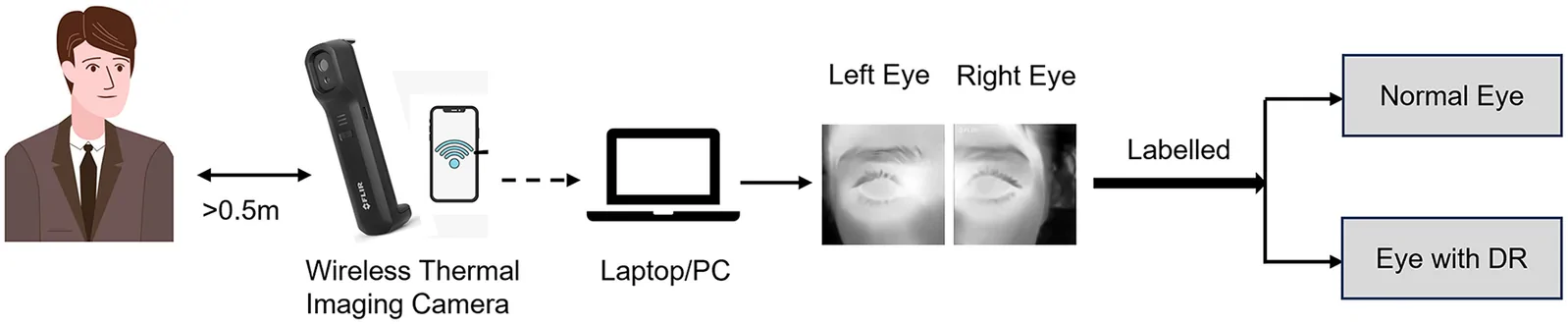
Data curation workflow for thermal eye image acquisition and labeling.

**Figure 3:**
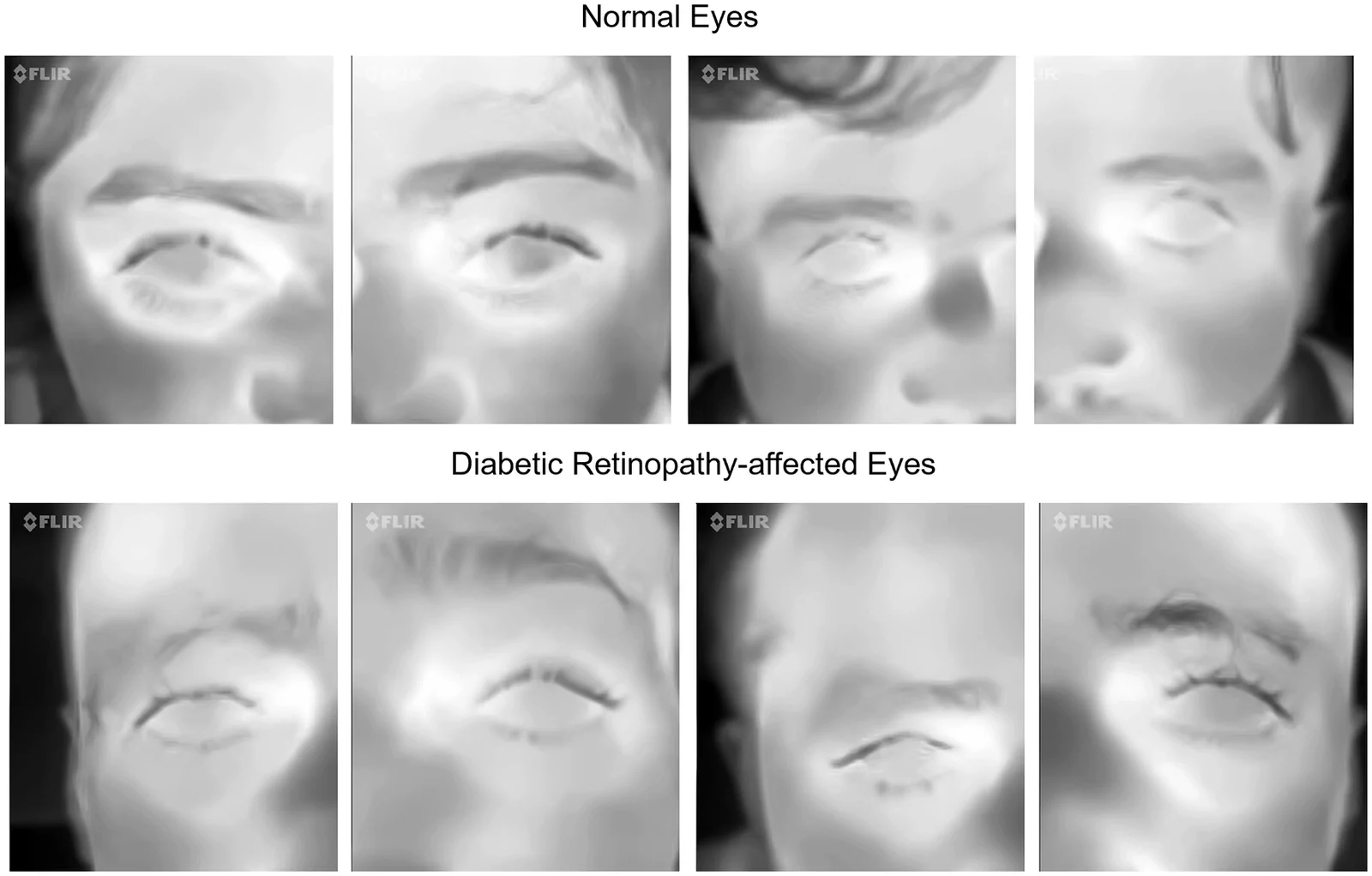
Sample images from the dataset.

A dedicated thermal eye dataset was developed at Aravind Eye Hospital, Chennai, using the FLIR Edge Pro wireless thermal camera. The camera provides a thermal resolution of 160 × 120 pixels (19,200 pixels), visual resolution of 640 × 480 pixels, thermal sensitivity of 70 mk, and a spectral range of 8–14 μm, operating between −20 °C and 120 °C. The data acquisition and curation workflow for thermal eye imaging is illustrated in Figure 2. Radiometric images were wirelessly transferred to an Android device and securely stored on a laptop for analysis. All imaging was carried out by trained engineers under medical supervision, following standard ophthalmic protocols. Participants were instructed to rest for 10 min before imaging and to avoid cosmetics, hot beverages, or eye drops. Only clinically confirmed normal and diabetic cases were included. Subjects with coexisting ocular comorbidities including dry eye, cataract, ocular surface disease, glaucoma, and recent topical medication use were excluded. Images were captured from a distance of about 0.5 m in a controlled environment maintained at 25 ± 1 °C. Each subject's eye was positioned to ensure full coverage while minimizing motion and temperature artifacts. The study adhered to the Declaration of Helsinki and received institutional ethics approval from Institutional Ethics Committee, Aravind Eye Hospital, Chennai (IEC-AEH CHN). Written informed consent was obtained from all participants; minors and non-consenting individuals were excluded. Each image was labeled and verified by two ophthalmologists and reviewed by a senior specialist, achieving an inter-rater reliability of κ = 0.87, indicating strong consistency. The diabetic group comprised patients with confirmed diabetic retinopathy on fundus examination; the normal group comprised healthy individuals with no diabetes or ocular pathology. Diabetic retinopathy was confirmed by fundus photography performed by a retinal specialist and graded using the international clinical diabetic retinopathy (ICDR) severity scale. Grading was performed independently by two fellowship-trained ophthalmologists, with discrepancies resolved by a senior retinal specialist, achieving an inter-rater reliability of κ = 0.87. The diabetic cohort included patients across all DR severity levels (mild to proliferative DR). Thermal eye images were acquired before pupil dilation under non-mydriatic conditions, and the clinical diagnosis from dilated fundus examination served as the patient-level reference standard for label assignment. Fundus graders were masked to thermal imaging data throughout the grading process. The normal group comprised healthy individuals with no diabetes or ocular pathology confirmed on clinical examination. The dataset summary is provided in Table 2, and representative thermal eye images from both normal and diabetic subjects of the collected data are shown in Figure 3.

**Table 2:** Summary of the thermal eye dataset curated for binary DR-vs.-normal classification.

Attribute	Details
Imaging device	FLIR edge pro thermal camera
Thermal resolution	160 × 120 pixels
Visual resolution	640 × 480 pixels
Thermal sensitivity	70 mk
Spectral range	8–14 μm
Temperature range	−20 °C and 120 °C
Color palette	Gray
Subjects	558 eye images (278 Normal, 280 eyes with DR)
Imaging distance	>0.5m
Ambient temperature	25 ± 1 °C
Gender	Male – 143, Female – 136
Age	25–60 years

Mk, milliKelvin; μm – micrometer; °C, degree Celsius.

#### 2.1.1 Image standardization

All thermal eye images were pre-processed to ensure consistency and suitability for model training. Each image was resized to a uniform resolution of 224 × 224 pixels and converted from a single-channel thermal format to pseudo-RGB by triplicating the grayscale channel, ensuring compatibility with pretrained CNN backbones. Intensity normalization was performed using the ImageNet mean and standard deviation values to align the thermal image distribution with the pretrained EdgeNeXt input space. Dataset splitting was performed group-wise by SubjectID to prevent data leakage between training and testing samples. Finally, class labels were encoded as *Normal* = *0* and *Eye with DR* = *1* for binary classification.

### 2.2 Temperature feature extraction and analysis

To quantitatively describe the thermal characteristics of ocular regions, a dedicated feature extraction workflow was developed using the Flyr radiometric interface (27). All analyses were performed on true temperature data (°C) rather than pseudo-colored intensity images, thereby preserving the physiological accuracy of the measurements. For each radiometric thermal image corresponding to either the left (LE) or right (RE) eye, pixel-level temperature matrices were decoded, normalized, and transformed into a standardized feature representation for subsequent statistical evaluation and multimodal integration. The decoded thermal matrix, denoted as (*x, y*), was restricted to a physical temperature range of 25 °C−40 °C, representing the physiologically relevant limits for periocular tissue (28). The clipped temperature matrix is represented as in Equation 1.


$$\begin{array}{l} T_{c}\left(x,y\right)=min\left(max\left(T\left(x,y\right),T_{\min}\right),T_{\max}\right) \end{array}$$ (1)

where $T_{\min}=25^{•}C$ and $T_{\max}=40^{•}C$. This constraint reduced the influence of sensor drift and environmental noise. The clipped temperature field was then quantized (*Q*_*T*_(*x, y*)) into 64 uniform thermal levels to ensure consistent computation of histograms and co-occurrence statistics across all samples. Equation 2 gives the quantized temperature levels.


$$\begin{array}{l} Q_{T}\left(x,y\right)=\left\lfloor \frac{\left(T_{c}\left(x,y\right)-T_{\min}\right)\times \left(L-1\right)}{T_{\max}-T_{\min}}\right\rfloor \end{array}$$ (2)

where *L* = 64 is the number of quantization levels. This normalization step enabled meaningful comparison between subjects by ensuring that observed variations reflected physiological differences rather than instrument-related artifacts.

#### 2.2.1 First-order temperature statistics

From the extracted temperature data, a comprehensive set of first-order statistical features was calculated to characterize the overall thermal behavior of each eye, in accordance with established approaches in ocular thermography research (18, 29, 30). The mean temperature (°C) ($\mu_{T}=\frac{1}{N}\overset{N}{\underset{i=1}{\sum }}T_{i}$) represents the average ocular surface temperature, reflecting baseline perfusion, while the standard deviation (°C) ($\sigma_{T}=\sqrt{\frac{1}{N}\overset{N}{\underset{i=1}{\sum }}{\left(T_{i}-\mu_{T}\right)}^{2}}$) quantifies the dispersion of temperature values, indicating variations in local heat distribution. The minimum and maximum (°C) values capture hypothermic and hyperthermic regions, respectively, and the temperature range (°C), defined as the difference between the maximum and minimum, expresses the overall spread of surface temperature. The coefficient of variation (CV), calculated as $\text{CV}=\frac{\sigma_{T}}{\mu_{T}}$, provides a normalized measure of variability relative to the mean. Higher-order moments, including skewness and kurtosis, describe the asymmetry and sharpness of the temperature distribution, which is given in Equations 3, 4.


$$\begin{array}{l} Skewness=\frac{1}{N}\overset{\;N}{\underset{i=1}{\sum }}{\left(\frac{T_{i}-\mu_{T}}{\sigma_{T}}\right)}^{3} \end{array}$$ (3)

$$\begin{array}{l} Kurtosis=\frac{1}{N}\overset{\;N}{\underset{i=1}{\sum }}{\left(\frac{T_{i}-\mu_{T}}{\sigma_{T}}\right)}^{4}-3 \end{array}$$ (4)

Together, these statistical measures form a concise numerical representation of the ocular thermal profile, enabling a clear distinction between the uniform temperature fields typically observed in healthy eyes and the irregular, localized variations associated with eyes with DR.

#### 2.2.2 GLCM-based spatial texture temperature metrics

To complement the statistical descriptors with spatial information, gray-level co-occurrence matrix (GLCM) features were computed directly from the quantized temperature maps, *Q*_*T*_(*x, y*) (31, 32). Let *P*(*i, j*; *d*, θ) denote the normalized co-occurrence probability that a pixel with gray level *i* occurs adjacent to a pixel with gray level *j* at distance *d*and orientation θ. The GLCM was generated for four orientations (0°, 45°, 90°, and 135°) and two-pixel distances (1 and 2) to capture both directional and neighborhood relationships among temperature levels. From each co-occurrence matrix, six Haralick texture measures were derived and averaged across orientations: contrast, representing the intensity of local temperature variations; homogeneity, indicating the smoothness of spatial temperature transitions; energy and angular second moment (ASM), describing the degree of regularity or repetition in thermal patterns; correlation, reflecting the linear dependency between neighboring temperature values; and dissimilarity, highlighting sharp thermal discontinuities. All these are expressed from Equations 5–9.


$$\begin{array}{l} Contrast=\overset{\;L}{\underset{i=1}{\sum }}\overset{\;L}{\underset{j=1}{\sum }}{\left(i-j\right)}^{2}P\left(i,j\right) \end{array}$$ (5)

$$\begin{array}{l} Homogeneity=\overset{\;L}{\underset{i=1}{\sum }}\overset{\;L}{\underset{j=1}{\sum }}\frac{P\left(i,j\right)}{1+|i-j|} \end{array}$$ (6)

$$\begin{array}{l} Energy=\overset{\;L}{\underset{i=1}{\sum }}\overset{\;L}{\underset{j=1}{\sum }}P{\left(i,j\right)}^{2} \end{array}$$ (7)

$$\begin{array}{l} Correlation=\frac{\sum_{i=1}^{\;L}\sum_{j=1}^{\;L}\left(i-\mu_{i}\right)\left(j-\mu_{j}\right)P\left(i,j\right)}{\sigma_{i}\sigma_{j}} \end{array}$$ (8)

$$\begin{array}{l} Dissimilarity=\overset{\;L}{\underset{i=1}{\sum }}\overset{\;L}{\underset{j=1}{\sum }}|i-j|P\left(i,j\right) \end{array}$$ (9)

Each feature can be averaged across all four orientations (0°, 45°, 90°, 135°) and distances *d* = 1, 2. These spatial texture features characterize the organization of temperature values across the ocular surface, offering a quantitative view of spatial uniformity and complexity.

For each thermal image, all extracted features were concatenated into a single composite vector encompassing global temperature descriptors, distribution-based statistics, and spatial texture metrics for integrated analysis. In total, 15 usable features were incorporated into the multimodal tabular branch after excluding metadata and class identifiers. These include temperature-based statistics such as mean, standard deviation, minimum, maximum, range, coefficient of variation, skewness, and kurtosis; and GLCM texture features comprising contrast, homogeneity, energy, correlation, angular second moment (ASM), and dissimilarity. The consolidated feature table included the filename, class label (Normal eyes with DR), subject identifier, and laterality (LE/RE). All results were exported to a structured CSV file, which served as the quantitative basis for subsequent multimodal analysis. All temperature-derived statistical and texture features were computed independently for each thermal image using its radiometric temperature matrix. Subject-wise group splitting was performed before model training, and all data-dependent preprocessing steps, including feature normalization, were applied on a fold-specific basis. The StandardScaler was fitted exclusively on training data within each fold and applied unchanged to validation or test samples, preventing information leakage.

### 2.3 Proposed Reliafusion-net: a reliability-aware hybrid method for normal eyes vs. eyes with diabetic retinopathy classification

**Figure 4:**
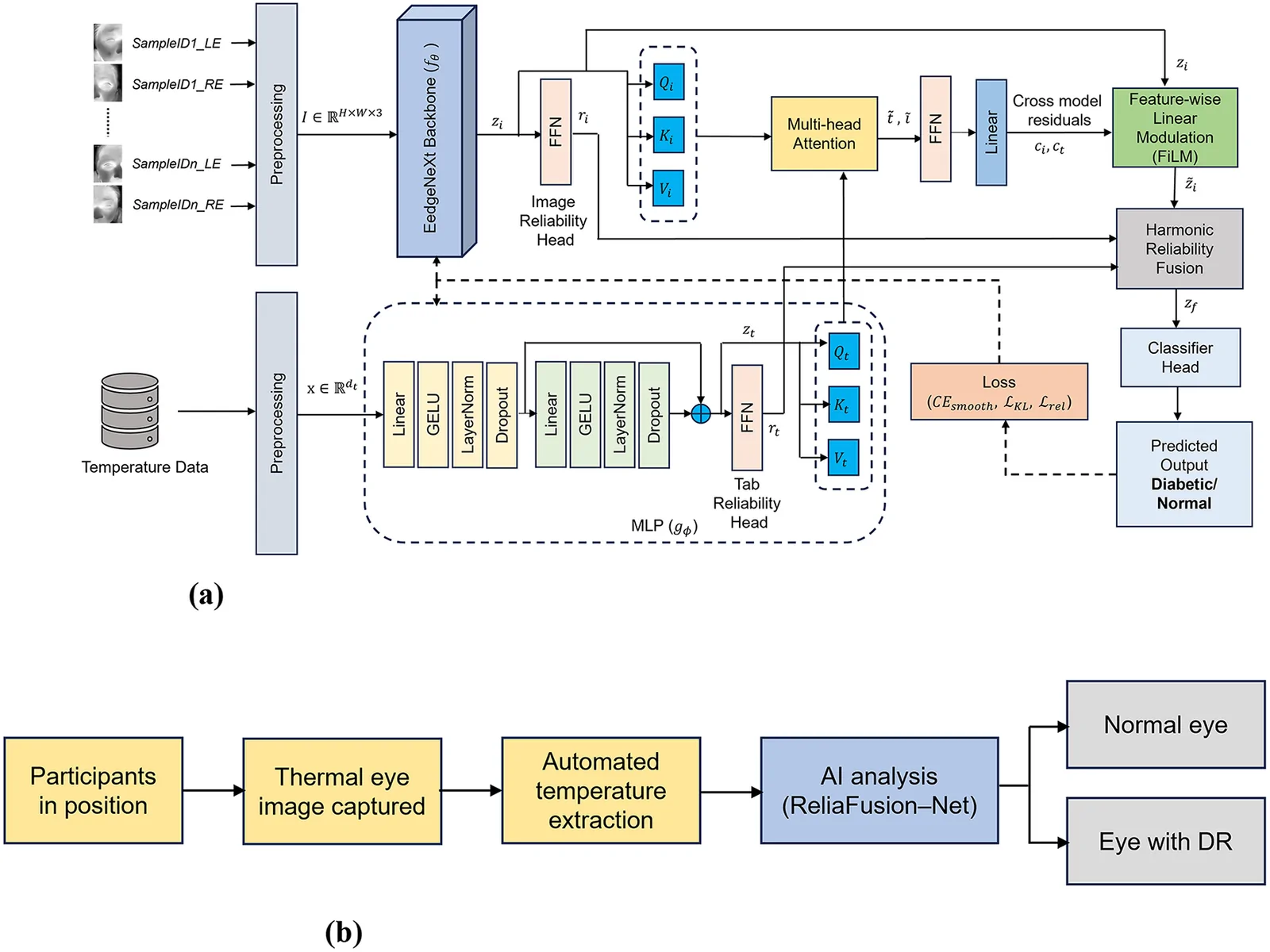
**(a)** Architecture of the proposed ReliaFusion-Net for normal eye vs. eye with DR classification. **(b)** Workflow of the proposed ReliaFusion-Net system.

The proposed ReliaFusion-Net jointly learns from thermal eye images and temperature data for robust diabetic-retinopathy classification. Unlike standard attention-based fusion methods that weight modalities based on feature similarity alone, ReliaFusion-Net introduces explicit per-sample reliability scores for each modality, supervised by a reliability alignment loss that ties each score to its branch's predictive confidence relative to the fused output—a distinction from fixed-weight, gating, and cross-attention approaches that lack this supervision-driven adaptivity. Figure 4a depicts the architecture of the proposed ReliaFusion-Net. Figure 4b shows the workflow steps of the proposed method. The architecture comprises two primary encoders, a deep convolutional image backbone and a lightweight tabular MLP branch followed by bidirectional co-attention, FiLM modulation, harmonic reliability gating, and multimodal fusion. The image backbone *f*_θ_ is based on a pretrained EdgeNeXt and takes a thermal eye image *I*∈ℝ^*H*×*W*×3^, where *H* and *W* represent the height and width of the image, respectively (the single-channel thermal frame is duplicated to three channels for CNN input). The backbone produces a global embedding ${\text{z}}_{i}=f_{\theta }\left(I\right)\in {\mathbb{R}}^{1280}$. The tabular MLP branch *g*_ϕ_ receives standardized temperature-based features $x\in {\mathbb{R}}^{d_{t}}$ (where *d*_*t*_ denotes the number of tabular features) and transforms them through successive fully connected layers with nonlinearity and normalization: *h*_1_ = *GELU*(*W*_1_*x*+*b*_1_), *h*_2_ = *LayerNorm*(*h*_1_), *h*_3_ = *Dropout*(*p*)*h*_2_, where *p* is the dropout probability. Here, *h*_1_, *h*_2_, and *h*_3_ represent intermediate hidden layer activations, *W* and *b* are the learnable weight and bias parameters, and *x* is the standardized tabular input vector. The Gaussian Error Linear Unit (GELU) activation introduces nonlinearity by weighting input values according to their probability under a Gaussian distribution, enabling smoother and probabilistically guided activation compared to ReLU. Layer Normalization (LayerNorm) normalizes activations across all hidden units within a layer, stabilizing gradients and improving convergence. After a residual feed-forward block of identical dimension *d* = 128, the final tabular embedding is given in Equation 10.


$$\begin{array}{l} z_{t}=g_{\phi }\left(x\right)=W_{2}h_{3}+b_{2} \end{array}$$ (10)

And the reliability head of the tabular stream is computed as given in Equation 11.


$$\begin{array}{l} r_{t}=\sigma \left(w_{r}^{⊤}GELU\left(W_{r}z_{t}+b_{r}\right)\right) \end{array}$$ (11)

Producing a scalar *r*_*t*_∈(0, 1) that quantifies tabular reliability. where σ(·) denotes the sigmoid activation function defined as σ(*x*) = 1/(1+*e*^−*x*^). The image branch computes an analogous embedding and reliability score as given in Equations 12, 13.


$$\begin{array}{l} z_{i}=f_{\theta }\left(I\right) \end{array}$$ (12)

$$\begin{array}{l} r_{i}=\sigma \left(w_{i}^{⊤}GELU\left(W_{i}z_{i}+b_{i}\right)\right) \end{array}$$ (13)

*z*_*t*_, *z*_*i*_ form the input tokens for cross-modal interaction, and each stream predicts its own reliability through a sigmoid gate, yielding *r*_*i*_ and *r*_*t*_, which represents the modality-specific confidence in its extracted representation (Equations 1–4). To enable cross-modal interaction, the image and tabular tokens (*z*_*i*_ and *z*_*t*_) are linearly projected into a shared embedding space using learnable projection matrices *W*_*q*_, *W*_*k*_, and *W*_*v*_, which generate query (Q), key (K), and value (V) representations for each modality. These projections allow both tokens to participate symmetrically in attention computation.

For the image token, the transformations are defined as in Equation 14:


$$\begin{array}{l} Q_{i}=W_{q}^{\left(i\right)}z_{i},K_{i}=W_{k}^{\left(i\right)}z_{i},V_{i}=W_{v}^{\left(i\right)}z_{i} \end{array}$$ (14)

And for the tabular token, the corresponding projections are in Equation 15:


$$\begin{array}{l} Q_{t}=W_{q}^{\left(t\right)}z_{t},K_{t}=W_{k}^{\left(t\right)}z_{t},V_{t}=W_{v}^{\left(t\right)}z_{t} \end{array}$$ (15)

here, each $W_{q}^{\left(\cdot \right)},W_{k}^{\left(\cdot \right)},W_{v}^{\left(\cdot \right)}\in {\mathbb{R}}^{d\times d_{h}}$ are learnable linear mappings that project the modality-specific tokens into a common latent space of dimension *d*_*h*_.

These projected tokens are subsequently passed through a Multi-Head Attention (MHA) mechanism, which enables bidirectional information exchange between modalities, allowing the tabular query to attend to image values and the image query to attend to tabular values. This interaction helps align complementary cues from visual and tabular domains within a shared latent space.

Formally, the multi-head attention operation computes a weighted combination of value vectors based on the scaled dot-product similarity between queries and keys. For the image–tabular pair, MHA is applied in both directions as given in Equation 16:


$$\begin{array}{l} \tilde{t}=MHA\left(Q_{t},K_{i},V_{i}\right),ĩ=MHA\left(Q_{i},K_{t},V_{t}\right) \end{array}$$ (16)

where each MHA block can be expressed as *MHA*(*Q, K, V*) = Concat(head_1_, …, head_*h*_)*W*_*O*_, Where *head* is represented using Equation 17.


$$\begin{array}{l} head_{j}=softmax\left(\frac{QW_{Q}^{\left(j\right)}{\left(KW_{K}^{\left(j\right)}\right)}^{⊤}}{\sqrt{d_{h}}}\right)VW_{V}^{\left(j\right)} \end{array}$$ (17)

with *h* denoting the number of attention heads, and $W_{Q}^{\left(j\right)},W_{K}^{\left(j\right)},W_{V}^{\left(j\right)},W_{O}$ being learnable parameters for each head. The resulting cross-attended representations $\tilde{t}\;$and ĩ are concatenated and refined through a feed-forward projection layer with nonlinearity and normalization to produce the cross-modal residuals *c*_*i*_and *c*_*t*_, as defined in Equation 18:


$$\begin{array}{l} \left[c_{i},c_{t}\right]=\Pi \left(W_{2}\text{GELU}\left(W_{1}\text{LayerNorm}\left(\left[\tilde{t}∥ĩ\right]\right)+b_{1}\right)+b_{2}\right) \end{array}$$ (18)

where $\left[\tilde{t}∥ĩ\right]$ denotes the concatenation of attended tabular and image features, and Π(·) represents the dimensional projection operator that maps them back to their respective modality feature spaces. In parallel, the co-attended tabular context *c*_*t*_ modulates the image embedding through Feature-wise Linear Modulation (FiLM). The FiLM block generates learnable gain and bias parameters as *g* = tanh(*W*_*g*_*c*_*t*_), *b* = tanh(*W*_*b*_*c*_*t*_), where *g* and *b* correspond to channel-wise scaling and shifting coefficients, respectively. Using these parameters, a channel-wise affine transformation is applied to the image representation to yield the FiLM-modulated image token given in Equation 19.


$$\begin{array}{l} {\tilde{z}}_{i}=z_{i}⊙\left(1+g\right)+b \end{array}$$ (19)

where ⊙ denotes element-wise multiplication. Next, a harmonic reliability gate fuses the co-attention-refined image feature (*z*_*i*_+*c*_*i*_) and the FiLM-modulated feature ${\tilde{z}}_{i}$ in proportion to their reliability scores. The reliability ratio ρ is computed as given in Equation 20.


$$\begin{array}{l} \rho =\frac{r_{i}}{r_{i}+r_{t}+\varepsilon } \end{array}$$ (20)

where *r*_*i*_ and *r*_*t*_ denote the reliability scalars of the image and tabular branches, and ε is a small constant for numerical stability. The adaptive fusion weight α^⋆^ is obtained as shown in Equation 21.


$$\begin{array}{l} \alpha^{⋆}=\sigma \left(\gamma \right)\rho +\left(1-\sigma \left(\gamma \right)\right)0.5 \end{array}$$ (21)

where σ(·) is the sigmoid activation and γ is a learnable scalar controlling the balance between adaptive and uniform weighting. The final fused token *z*_*f*_ combines both components harmonically as given in Equation 22 ensuring that the contribution of each modality is proportional to its estimated reliability.


$$\begin{array}{l} z_{f}=\alpha^{⋆}\left(z_{i}+c_{i}\right)+\left(1-\alpha^{⋆}\right){\tilde{z}}_{i} \end{array}$$ (22)

Finally, modality-specific classification heads map each latent representation to its corresponding logit vector as in Equation 23.


$$\begin{array}{l} \ell_{fuse}=W_{f}z_{f},\ell_{img}=W_{i}z_{i},\ell_{tab}=W_{t}z_{t} \end{array}$$ (23)

which are subsequently used for joint optimization and reliability-aware prediction. The total training loss combines three components: agreement (co-distillation) between fused and unimodal predictions (Equation 24), reliability alignment, which encourages reliability scores to track each branch's accuracy (Equation 26), and label-smoothed cross-entropy on the fused logits (Equation 29).

The agreement loss enforces consistency between the fused and unimodal outputs and is formulated as


$$\begin{array}{l} L_{\text{KL}}=\text{KL}\left(\text{softmax}\left(\ell_{\text{fuse}}\right)∥\text{softmax}\left(\ell_{\text{img}}\right)\right)\\ \;+\;\text{KL}\left(\text{softmax}\left(\ell_{\text{fuse}}\right)∥\text{softmax}\left(\ell_{\text{tab}}\right)\right) \end{array}$$ (24)

where the Kullback–Leibler (KL) divergence measures how one probability distribution *P* diverges from another distribution *Q*. In this context, *P* = softmax(ℓ_*fuse*_) and *Q* = softmax(ℓ_*img*_)or softmax(ℓ_*tab*_). Formally KL is given in Equation 25,


$$\begin{array}{l} \text{KL}\left(P∥Q\right)=\overset{C}{\underset{c=1}{\sum }}P_{c}\log\frac{P_{c}}{Q_{c}} \end{array}$$ (25)

If the fused and unimodal branches predict similar class probabilities, KL(*P*∥*Q*) becomes small. Minimizing this term promotes agreement and knowledge transfer from the fused output to each unimodal stream, stabilizing multimodal learning. Because KL divergence is asymmetric, the fused distribution is treated as the reference.

The reliability alignment loss $L_{rel}\;$ensures that each modality's reliability score reflects its relative predictive performance:


$$\begin{array}{l} L_{\text{rel}}=\text{BCE}\left(r_{i},1\;\left[\text{CE}\left(\ell_{\text{img}}\right)<\text{CE}\left(\ell_{\text{fuse}}\right)\right]\right)\\ \;+\;\text{BCE}\left(r_{t},1\;\left[\text{CE}\left(\ell_{\text{tab}}\right)<\text{CE}\left(\ell_{\text{fuse}}\right)\right]\right) \end{array}$$ (26)

where Binary Cross-Entropy (BCE) measures the difference between a predicted reliability score (*r*_*i*_ or *r*_*t*_) and a binary target. For a single sample, BCE is given as in Equation 27,


$$\begin{array}{l} \text{BCE}\left(r,y_{\text{target}}\right)=-\left[y_{\text{target}}\log\left(r\right)+\left(1-y_{target}\right)\log\left(1-r\right)\right] \end{array}$$ (27)

When a branch (image or tabular) achieves lower cross-entropy loss, it is assigned a target of 1, signifying higher reliability. This alignment mechanism encourages adaptive weighting of modalities according to their relative predictive confidence.

The label-smoothed cross-entropy loss $L_{\text{CE}}$ replaces hard one-hot targets with slightly softened distributions to prevent overconfidence. The standard cross-entropy is defined as in Equation 28.


$$\begin{array}{l} \text{CE}\left(\ell_{\text{fuse}},y\right)=-\log\frac{\exp\left(\ell_{\text{fuse},y}\right)}{\sum_{c=1}^{C}\exp\left(\ell_{\text{fuse},c}\right)} \end{array}$$ (28)

With label smoothing (parameter ε), the target distribution becomes


$$y_{c}^{\text{smooth}}=\{\begin{array}{c} 1-\varepsilon ,\;c=y,\\ \frac{\varepsilon }{C-1},\;c\neq y, \end{array}$$

and the smoothed cross-entropy is


$$\begin{array}{l} {\text{CE}}_{\text{smooth}}=-\overset{C}{\underset{c=1}{\sum }}y_{c}^{\text{smooth}}\log\frac{\exp\left(\ell_{\text{fuse},c}\right)}{\sum_{k=1}^{C}\exp\left(\ell_{\text{fuse},k}\right)} \end{array}$$ (29)

Label smoothing mitigates overconfidence (probabilities near 1.0) and improves model calibration. It serves as the primary classification loss for the fused output.

The overall loss function combines all three components as in Equation 30.


$$\begin{array}{l} L=CE_{\text{smooth}}\left(\ell_{\text{fuse}},y\right)+\lambda_{\text{KL}}L_{\text{KL}}+\lambda_{\text{rel}}L_{\text{rel}} \end{array}$$ (30)

where λ_KL_ and λ_rel_ are weighting factors balancing the auxiliary losses.

Optimization is performed using AdamW with a cosine-annealed learning rate schedule and exponential moving average (EMA) updates. The image backbone is initially frozen during warm-up epochs and later unfrozen for joint training.

During inference, calibrated probabilities are produced through temperature scaling and an optional per-class bias adjustment as (Equation 31).


$$\begin{array}{l} \hat{\ell }=\frac{\ell_{\text{fuse}}}{T},{\hat{\ell }}^{\prime }=\hat{\ell }-\tau ,\hat{p}=\text{softmax}\left({\hat{\ell }}^{\prime }\right) \end{array}$$ (31)

where *T* denotes the temperature parameter and τ represents class-wise bias offsets used for *post-hoc* probability calibration. Finally yields a reliability-aware multimodal decision framework where the convolutional backbone extracts spatial cues, the MLP encodes tabular statistics, and ReliaFusion-Net fuses them for robust disease classification. The proposed reliability mechanism is trained under clinically curated input conditions, where degraded or noisy thermal images are excluded during ophthalmologist-led verification and missing tabular values are systematically handled during preprocessing. Accordingly, the learned reliability scores represent relative, sample-adaptive modality confidence under realistic clinical data quality.

### 2.4 Model evaluation and performance metrics

The model performance is quantitatively assessed using standard classification and threshold-free metrics. Overall accuracy is defined as in Equation 32.


$$\begin{array}{l} Accuracy=\frac{TP+TN}{TP+TN+FP+FN} \end{array}$$ (32)

where *TP*, *TN*, *FP*, and *FN* denote true positives, true negatives, false positives, and false negatives, respectively. The precision or positive predictive value (PPV), recall, specificity, and negative predictive value (NPV) are computed as (Equations 33–36).


$$\begin{array}{l} Precision\;\left(PPV\right)=\frac{TP}{TP+FP} \end{array}$$ (33)

$$\begin{array}{l} Recall\;\left(Sensitivity\right)=\frac{TP}{TP+FN} \end{array}$$ (34)

$$\begin{array}{l} Specificity=\;\frac{TN}{TN+FP} \end{array}$$ (35)

$$\begin{array}{l} NPV=\;\frac{TN}{TN+FN} \end{array}$$ (36)

and their harmonic mean, the F1-score, is expressed as (Equation 37).


$$\begin{array}{l} F1=2\cdot \frac{Precision\cdot Recall}{Precision+Recall} \end{array}$$ (37)

To evaluate discrimination capability independent of classification thresholds, the receiver operating characteristic (ROC) curve and its area under curve (AUC), as well as the precision–recall (PR) curve and average precision (AP), are employed. Additional evaluation metrics were employed to assess model performance and learning behavior. Log loss was used to measure probabilistic calibration, while confusion matrices provided a visual summary of class-wise prediction outcomes. Model efficiency was further examined through parameter count, GFLOPs, model size, and inference latency. In addition, epoch-wise plots of loss, accuracy, F1-score, and AUC were analyzed to illustrate the learning progression and overall training stability.

## 3 Results and discussion

The ReliaFusion-Net framework was implemented in Python and executed on Google Colab using an NVIDIA T4 GPU to accelerate model training. The code was developed and optimized on a Dell laptop equipped with an Intel Core i5 processor, where all pre-processing and post-analysis (data preparation, visualization, and result interpretation) were performed.

### 3.1 Statistical analysis of temperature data

**Figure 5:**
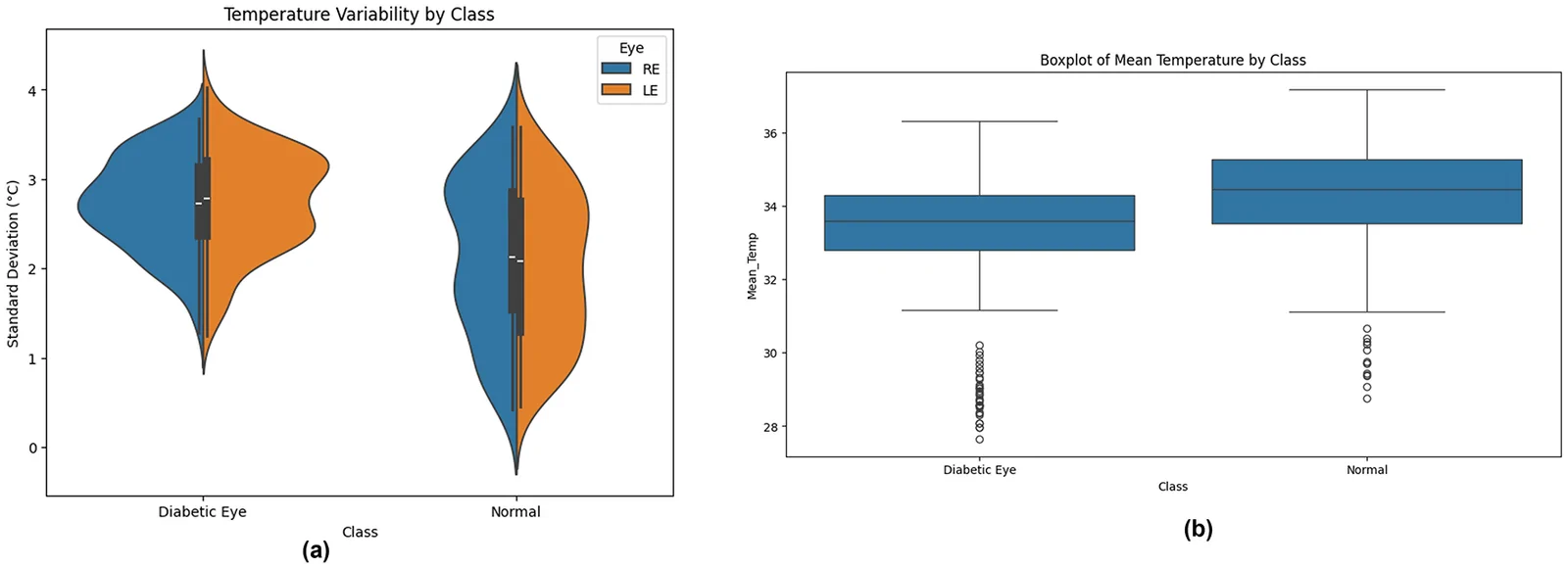
**(a)** Violin plot of *Std_Temp* by class & eye—Visualizes intra-class variability (important physiological cue). **(b)** Boxplot of *Mean_Temp* by class—Highlights greater temperature variability in eye with DR.

**Figure 6:**
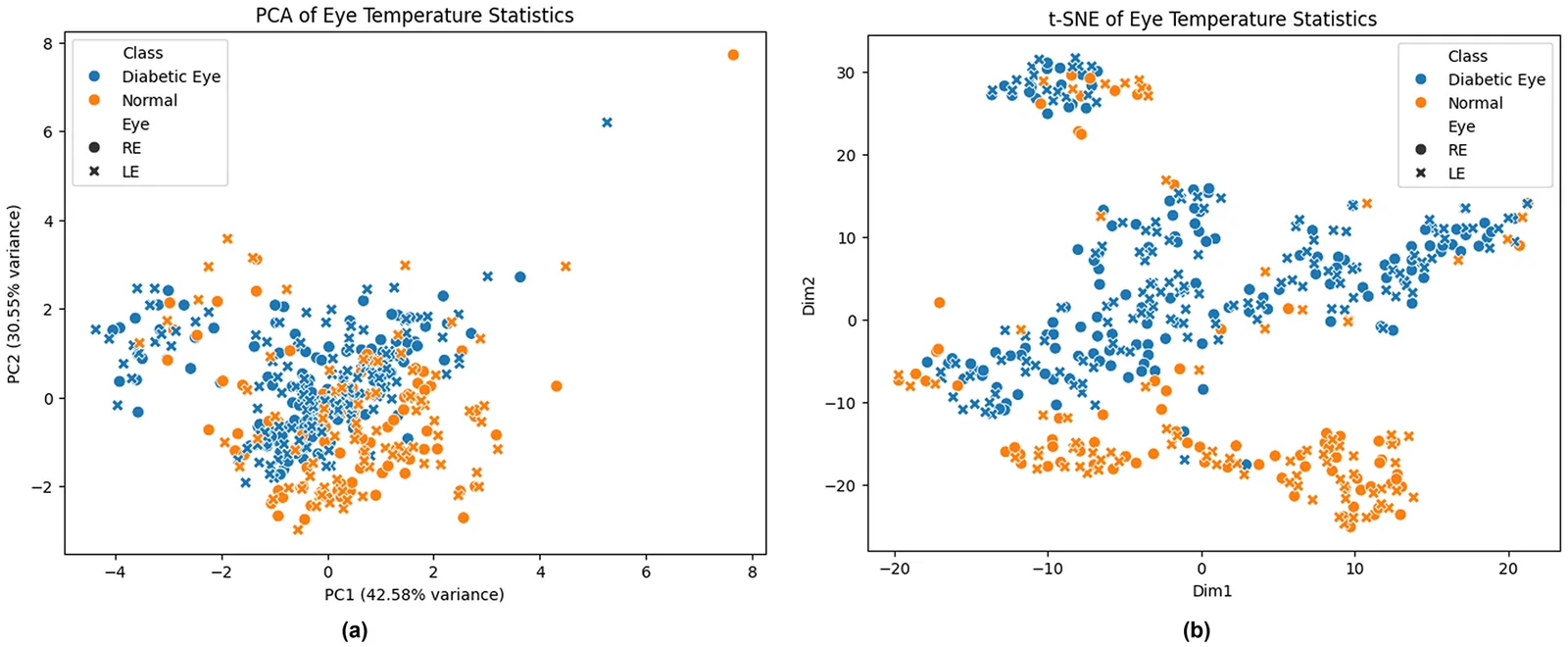
**(a)** PCA plot—Illustrates linear separability and feature compactness. **(b)** t-SNE—Demonstrates nonlinear separability and class clustering strength.

To examine thermal variations between normal and eyes with DR, descriptive and inferential analyses were performed on temperature-derived statistical features. The violin and box plots (Figures 5a, b) indicate that, although normal eyes exhibit higher absolute mean temperatureseyes with DR demonstrate broader temperature distributions and increased variability, reflecting elevated thermal asymmetry and localized instability associated with microvascular alterations. The scatter matrix and dimensionality-reduction projections (PCA and t-SNE, Figures 6a, b) show a clear separation between the two classes, confirming the discriminative potential of the temperature features. Tukey's HSD *post-hoc* analysis (Table 3) revealed statistically significant differences (*p* < 0.001) across most temperature-related features, including higher *mean* (+1.0067 °C) and *minimum* (+3.00 °C) temperatures in Normal eyes compared to eyes with DR. Eyes with DR exhibited greater temperature variability (−0.67 °C in *Std_Temp*), while *Max_Temp* showed no significant difference (*p* = 0.26). Moreover, Normal eyes displayed slightly higher *skewness* (+0.29) and lower *kurtosis* (−0.58), indicating a more right-skewed and flatter thermal distribution. Overall, the thermal metrics reflect consistent physiological distinctions between healthy and pathological ocular states, validating their use as discriminative features for Normal vs. eyes with DR classification.

**Table 3:** *Post-hoc* Tukey HSD comparison of temperature statistics between eyes with DR and normal eyes.

Feature	Mean difference (normal – eye with DR)	95% CI (lower–upper)	*p*-value	Significance
Mean_temp (°C)	+1.0067	0.6429–1.3704	< 0.001	✔ Significant
Std_temp (°C)	−0.6746	−0.8068–−0.5423	< 0.001	✔ Significant
Min_temp (°C)	+3.0184	2.6981–3.3386	< 0.001	✔ Significant
Max_temp (°C)	+0.2095	−0.1557–0.5746	0.2601	✖ Not Significant
Skewness	+0.291	0.1925–0.3896	< 0.001	✔ Significant
Kurtosis	−0.586	−0.985–−0.187	< 0.001	✔ Significant

Positive mean difference indicates higher values in normal subjects, while negative values indicate higher values in eye with DR subjects. CI, confidence interval; °C, degree Celsius.

A comprehensive temperature and texture-based feature analysis was conducted to quantify inter-class (Normal vs. eye with DR) and inter-ocular (LE vs. RE) variations in thermal eye images. As summarized in Table 4, although Normal eyes exhibited higher absolute mean and percentile-based temperature values, eyes with DR demonstrated markedly greater thermal variability, dispersion, and textural irregularity. Specifically, eyes with DR showed substantially lower minimum temperatures and a wider temperature range, accompanied by increased standard deviation (Std ≈ 2.75 °C for eye with DR vs. 1.92 °C for Normal), indicating enhanced thermal instability. Coefficient of variation values were also higher in eyes with DR (0.08 ± 0.02 vs. 0.06 ± 0.02), further supporting increased dispersion. Distributional descriptors such as skewness and kurtosis revealed more asymmetric and flatter temperature distributions in eyes with DR, consistent with heterogeneous thermal behavior. Entropy was elevated in eyes with DR (4.76 ± 0.27 vs. 4.39 ± 0.42), reflecting increased disorder in the thermal field. GLCM-based texture features reinforced this observation, with higher contrast and dissimilarity and reduced energy, ASM, and homogeneity in eyes with DR, indicating rougher and less uniform thermal textures.

**Table 4:** Summary of temperature and GLCM-based textural statistics for Normal and eyes with DR.

Feature	LE (eye with DR)	RE (eye with DR)	LE (normal)	RE (normal)	Observation
Std (°C)	2.77 ± 0.55	2.73 ± 0.50	1.90 ± 0.63	1.94 ± 0.65	Eye with DR shows higher variability
Min temp (°C)	26.21 ± 0.85	26.29 ± 0.86	29.18 ± 1.88	29.32 ± 1.78	Lower minima in eye with DR
Range (°C)	10.66 ± 1.50	10.61 ± 1.25	7.76 ± 1.81	7.54 ± 1.73	Larger range for eye with DR
CoeffVar	0.08 ± 0.02	0.08 ± 0.01	0.06 ± 0.02	0.06 ± 0.02	Higher dispersion in eye with DR
Entropy	4.76 ± 0.27	4.76 ± 0.25	4.39 ± 0.42	4.38 ± 0.42	Increased irregularity in eye with DR
GLCM contrast	0.20 ± 0.04	0.20 ± 0.04	0.16 ± 0.06	0.15 ± 0.06	Eye with DR images more textured
GLCM homogeneity	0.92 ± 0.01	0.92 ± 0.01	0.93 ± 0.02	0.93 ± 0.02	Lower homogeneity in eye with DR
GLCM energy	0.20 ± 0.03	0.20 ± 0.03	0.24 ± 0.04	0.24 ± 0.04	Lower uniformity in eye with DR
GLCM ASM	0.04 ± 0.01	0.04 ± 0.01	0.06 ± 0.02	0.06 ± 0.02	Eyes with DR less structured
GLCM dissimilarity	0.16 ± 0.02	0.16 ± 0.02	0.14 ± 0.05	0.14 ± 0.04	Greater textural contrast in eye with DR

Eyes with DR exhibit higher thermal variability and greater textural irregularity compared to normal eyes. LE, left eye; RE, right eye; GLCM, gray level co-occurrence matrix; ASM, angular second moment; CoeffVar, coefficient of variation; °C, degree Celsius.

Bilateral analysis of left and right eyes showed comparable trends, suggesting symmetric disease-related alterations. Overall, thermal and textural parameters consistently delineate eyes with DR as exhibiting greater thermal instability and rougher temperature textures.

The age distribution differed between groups, with the normal cohort having a mean age of 40.6 ± 11.4 years and the diabetic cohort 51.7 ± 6.6 years (range: 26–60 years). This disparity reflects the well-established epidemiology of diabetic retinopathy, which disproportionately affects older individuals with longer disease duration and is characteristic of real-world ocular screening populations. To assess whether age confounds the observed thermal feature associations, an age-adjusted sensitivity analysis was conducted using partial correlations and age-adjusted logistic regression, controlling for age as a covariate. Results are presented in Table 5. All features except Max Temperature remained highly significant (*p* < 0.001) following age adjustment; Max Temperature was non-significant before adjustment as well (Tukey's HSD *post-hoc* analysis, Table 3), indicating this is not attributable to age. Notably, partial correlation coefficients for the majority of features strengthened post-adjustment, indicating that age acts as a suppressor rather than an inflator of the thermal signal—suggesting the true discriminative power of thermal features may be conservatively estimated in the unadjusted analysis.

**Table 5:** Age-adjusted sensitivity analysis of ocular thermal features.

Features	r_raw	p_raw	r_partial	p_partial	Significance
Mean temperature	−0.2546	9.00e−08	−0.2620	3.63e−08	^***^
Std. deviation	0.4365	2.20e−21	0.5210	3.20e−31	^***^
Min temperature	−0.6675	1.17e−56	−0.7134	6.11e−68	^***^
Max temperature	−0.0545	2.60e−01	−0.0327	4.99e-01	NS
Temperature range	0.6065	1.93e−44	0.6613	2.85e−55	^***^
Coefficient of variation	0.4658	1.74e−24	0.5427	3.19e−34	^***^
Skewness	−0.2705	1.25e−08	−0.1965	4.17e−05	^***^
Kurtosis	0.1384	4.09e−04	0.0560	2.47e−04	^***^
Entropy	0.4174	1.62e−19	0.5632	2.84e−37	^***^
GLCM contrast	0.3747	9.54e−16	0.4542	3.20e−23	^***^
GLCM homogeneity	−0.3633	7.90e−15	−0.4957	5.53e−28	^***^
GLCM energy	−0.3714	1.79e−15	−0.4986	2.40e−28	^***^
GLCM ASM	−0.3823	2.23e−16	−0.5264	6.05e−32	^***^
GLCM dissimilarity	0.3695	2.52e−15	0.4913	1.87e−27	^***^

r_raw, unadjusted correlation coefficient; p_raw, p-value of unadjusted correlation; r_partial, partial correlation coefficient controlling for age; p_partial, p-value of partial correlation; NS, not significant; ^***^p < 0.001.

### 3.2 Performance analysis of the proposed ReliaFusion-net

Table 6 summarizes the key hyperparameters used in model training. The network was trained for 40 epochs with a batch size of 16, employing an initial learning rate of 1 × 10^−4^ for the fusion head and 3 × 10^−5^ for the backbone, using the AdamW optimizer with a cosine annealing schedule. Label smoothing (ε = 0.02) and exponential moving average (β_ema_ = 0.999) were applied for regularization and stability, and early stopping was enabled with a patience of 10 epochs to avoid overfitting. In the ReliaFusion-Net framework, each input sample consists of one image and its corresponding tabular feature vector representing the same subject eye pair (e.g., *SubjectID _1 LE*). The dataset is split into training, validation, and test sets using *GroupShuffleSplit* based on the SubjectID, ensuring that the same subject never appears in more than one split, thereby preventing data leakage. Each record in the DataFrame links an image path, tabular features, and class label, so during model training and evaluation, the dataloader feeds the network with correctly paired image–tabular inputs in a 1-to-1 manner for consistent multimodal learning. For the tabular branch, the specified feature columns were processed through a deterministic cleaning-and-scaling pipeline. All selected columns were coerced to numeric using pd.to_numeric (errors=“coerce”), converting invalid entries to NaN. Missing values were imputed with the column median, and any remaining NaN or infinite values (±inf) were replaced with 0.0 to ensure numerical stability. The resulting matrix was cast to float32, reshaped as needed, and standardized using a pre-trained StandardScaler (zero mean, unit variance) trained on the training split. Initially, the dataset was divided into 80% for training + validation and 20% for testing. The training + validation subset was then further split into 80% training and 20% validation, resulting in an overall ratio of approximately 64:16:20 for train, validation, and test sets, respectively.

**Table 6:** Hyperparameter configuration of the proposed ReliaFusion-Net framework.

Parameter	Value/description
Batch size	16
Epochs	40
Warm-up epochs	8 (backbone frozen)
Learning rate (head/backbone)	1 × 10^−4^/3 × 10^−5^
Weight decay	1 × 10^−4^
Shared attention dim	256
Attention heads	4
MLP Hidden dim/dropout	128/0.3
Image backbone architecture	EdgeNeXt
Img size	224 × 224
Data split	Group-wise by SubjectID

MLP, multi-layer perceptron; Img, Image.

**Figure 7:**
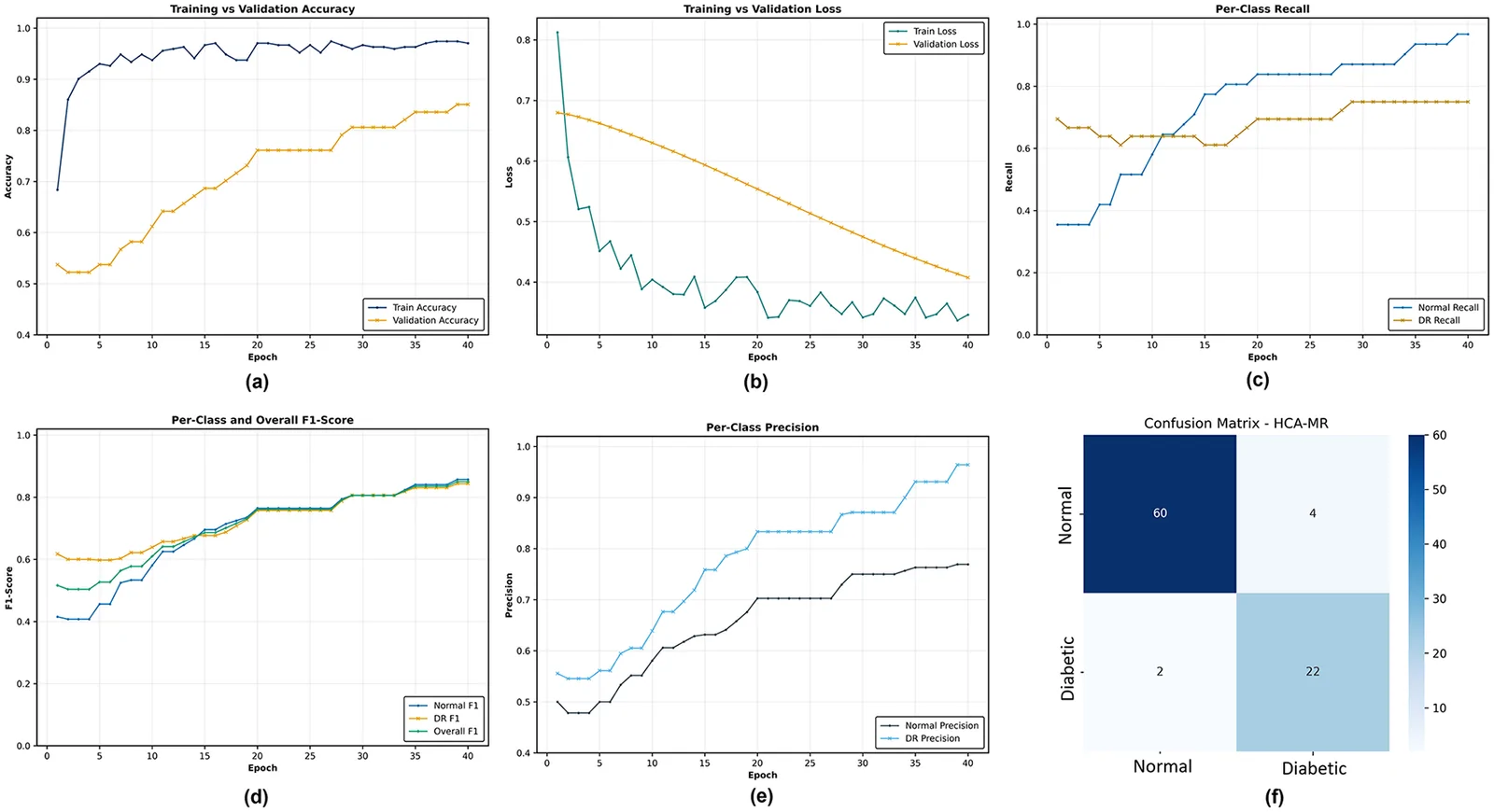
Training and validation performance curves of the proposed ReliaFusion-Net: **(a)** training vs. validation accuracy, **(b)** training vs. validation loss, **(c)** per-class and overall F1-score, **(d)** per-class precision, **(e)** per-class recall, and **(f)** Confusion matrix.

The proposed ReliaFusion-Net performed exceptionally well on the test set, achieving an overall accuracy of 93.18%, with precision, recall, and F1-score as 93.46%, 93.18%, 93.26% and an AUC of 97.85%. These results demonstrate the model's strong ability to distinguish between classes and its consistent performance across data samples. Figure 7 presents detailed training and validation dynamics. The accuracy and AUC curves (Figures 7a, b) show rapid and stable convergence without overfitting. Class-wise metrics (Figures 7c–e) indicate consistent improvement of both classes (Normal and DR), validating the balanced learning behavior of the fusion network. The confusion matrix (Figure 7f) demonstrates excellent classification performance on the held-out test.

**Figure 8:**
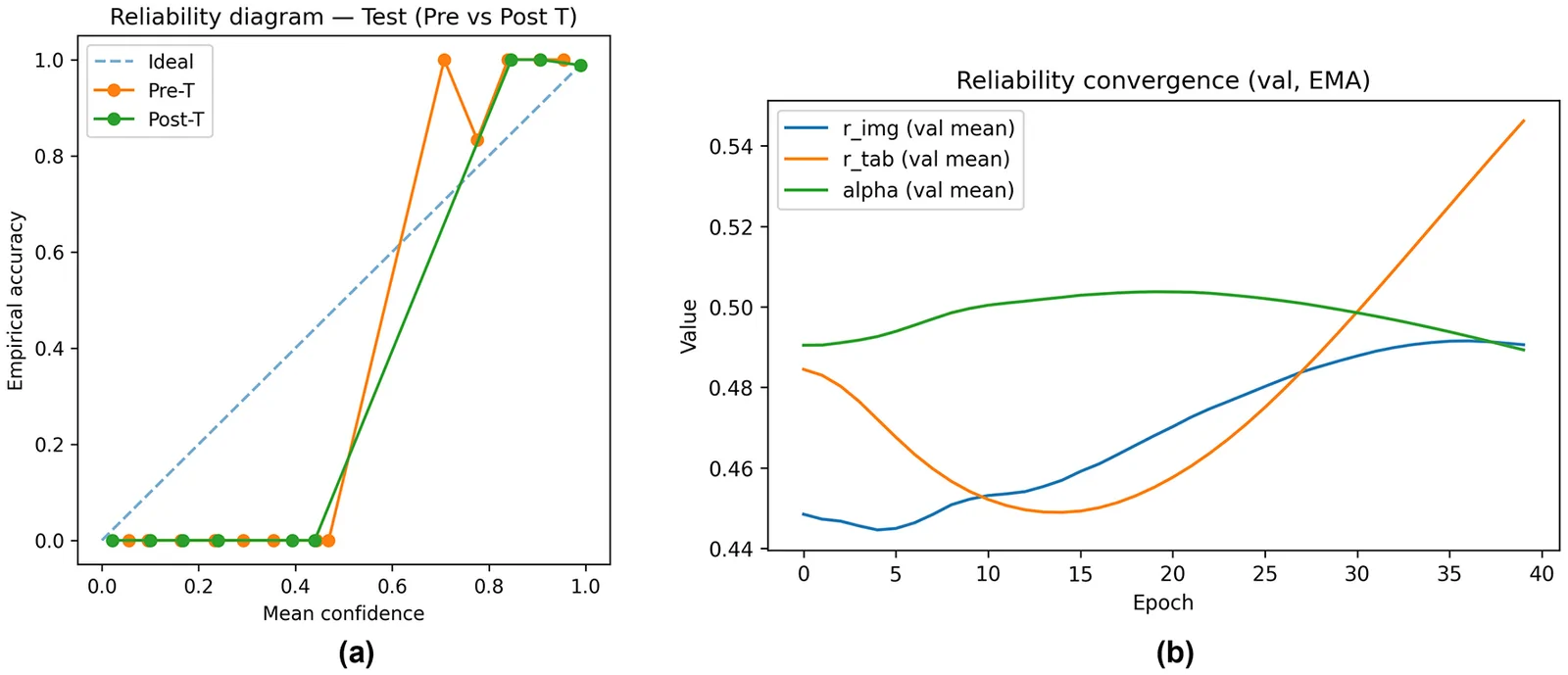
Reliability and calibration analysis of the ReliaFusion-Net: **(a)** improved post-calibration alignment, **(b)** convergence of modality reliabilities and fusion weight.

In addition to accuracy, the model's reliability across modalities was examined, as shown in Figure 8. Figure 8a shows the reliability diagram comparing calibration before and after temperature scaling. The pre-temperature (Pre-T) curve is slightly overconfident, while the post-temperature (Post-T) curve aligns more closely with the ideal diagonal, confirming improved confidence calibration. Figure 8b illustrates the validation-phase convergence of image reliability (*r*_*img*_), tabular reliability (*r*_*tab*_), and the adaptive fusion weight (α), showing stable learning where both modalities reach comparable reliability levels. Table 7 evaluates ReliaFusion-Net robustness under corrupted input conditions. Under clean conditions, fusion AUC (0.9785) substantially outperformed image-only (0.7630) and tabular-only (0.8353) baselines. Under Gaussian noise and tabular masking, fusion AUC declined marginally (0.9556–0.9720), with reduced *r*_*img*_ values confirming adaptive down-weighting of corrupted modalities. In missing modality scenarios, image-missing caused minimal degradation (AUC 0.9700), while tabular-missing resulted in a larger drop (AUC 0.8066). Across all conditions, the fusion model consistently outperformed unimodal baselines, validating the reliability-aware fusion strategy.

**Table 7:** Robustness evaluation of ReliaFusion-Net under varying corruption types and severity levels.

Corruption type	Severity	Fusion (AUC)	Image-only	Tabular-only	Mean r_*img*_	Mean r_*tab*_	Mean α
Clean	–	0.9785	0.7630	0.8353	0.4506	0.4801	0.5012
Gaussian noise	0.05	0.9647	0.7470	0.8353	0.4060	0.4396	0.4907
Gaussian noise	0.1	0.9556	0.7240	0.8353	0.3996	0.4396	0.4887
Tab mask	0.1	0.9720	0.7630	0.8350	0.3806	0.4446	0.4816
Tab mask	0.25	0.9675	0.7630	0.7733	0.3806	0.4417	0.4823
Missing modality	image missing	0.9700	0.6667	0.8353	0.4214	0.4396	0.4958
Missing modality	tab missing	0.8066	0.7630	0.5000	0.3806	0.4117	0.4899

AUC, area under the curve; *r*_*img*_, image modality reliability score; *r*_*tab*_, tabular modality reliability score; α, adaptive fusion weight; Tab mask, tabular feature masking; Clean, no corruption applied.

**Figure 9:**
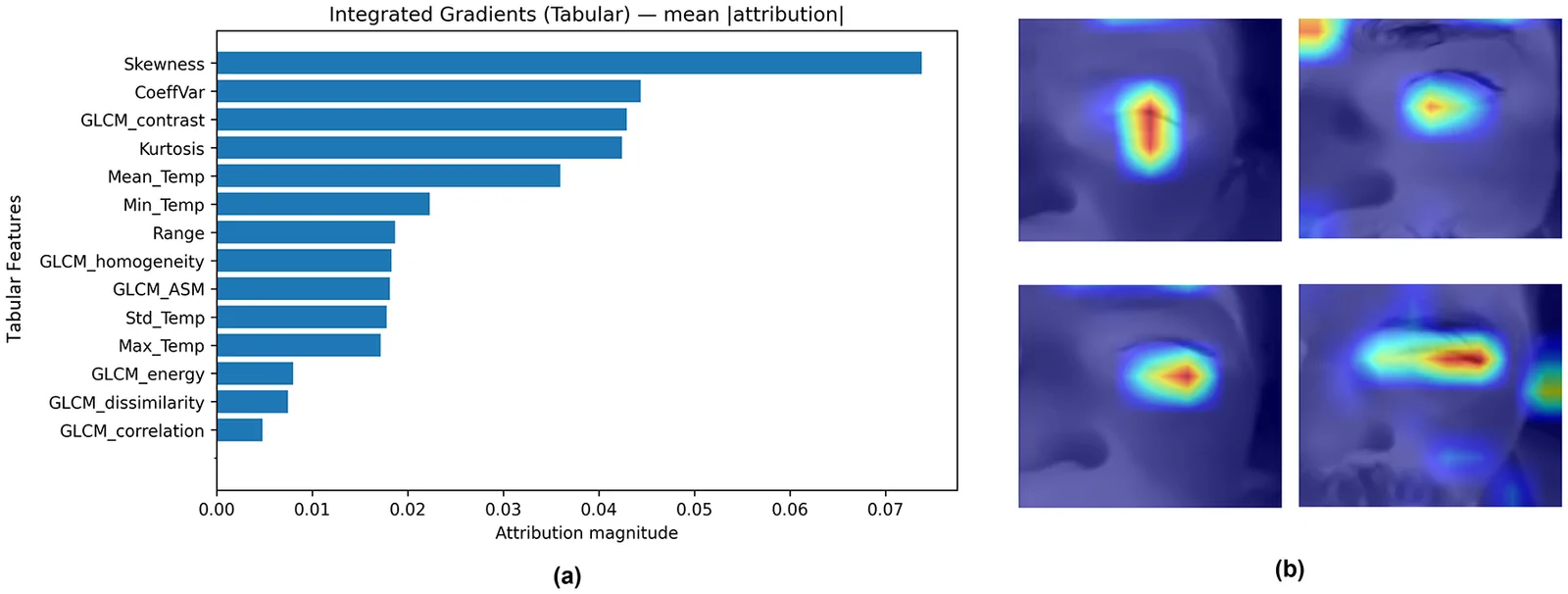
Interpretability analysis of the proposed ReliaFusion-Net model. **(a)** Integrated Gradients feature importance. **(b)** Grad-CAM visualizations.

The integrated gradient plot (Figure 9a) illustrates the relative contribution of tabular features to the model's prediction by integrating the gradients of the output with respect to each feature. Features such as Skewness, CoeffVar, and GLCM_contrast exhibit the highest attribution magnitudes, indicating their dominant influence on classification. High attribution of skewness and coefficient of variation reflects asymmetric periocular temperature distributions, while elevated GLCM_contrast attributions suggest that thermal texture irregularities associated with localized perfusion changes contribute meaningfully to classification. These findings show that both temperature-derived statistical measures and texture-based descriptors play critical roles in decision-making. The Grad-CAM heatmaps shown in Figure 9b highlight the key regions in the thermal eye images that most strongly influenced the model's predictions. The activations are primarily concentrated around the eyelid, tear duct, and periorbital regions, corresponding to physiologically relevant zones of temperature variation. Activation in the eyelid margin, tear duct, and periorbital regions aligns with documented ocular surface temperature alterations and microvascular perfusion changes associated with diabetic retinopathy. These visual explanations demonstrate that the tabular and image-based components contribute in complementary ways: integrated gradient (IG) analysis identifies which features are most influential, while Grad-CAM localizes the regions of visual focus. Together, they enhance the transparency and interpretability of the ReliaFusion-Net framework.

### 3.3 Ablation study and cross-validation performance

To evaluate the contribution of each component within the proposed framework, an ablation study was conducted (Table 8). When only image and temperature modalities were used independently, accuracies of 71.36 and 85.05% were obtained, respectively. Removing the bi-directional co-attention or FiLM modules led to noticeable reductions in performance, confirming their synergistic role in cross-modal alignment. In contrast, combining both modalities via ReliaFusion-Net achieved the best overall performance (Accuracy = 93.18%, F1 = 93.26%, AUC = 97.85%). To further validate the proposed fusion strategy, ReliaFusion-Net was compared against two baseline fusion approaches using both modalities. The fixed-weight fusion method achieved an accuracy of 90.91% and AUC of 0.9623, while the feature concatenation method achieved an accuracy of 90.91% and AUC of 0.9653. ReliaFusion-Net outperformed both baselines, demonstrating that reliability-aware adaptive fusion yields superior classification performance. Table 9 presents the classification performance of ReliaFusion-Net on the held-out test set. The model achieved a sensitivity of 93.18% and specificity of 93.75%, with a high NPV of 96.77%, indicating reliable identification of normal eyes. All metrics are reported with 95% confidence intervals, reflecting estimation reliability given the modest test set size.

**Table 8:** Ablation results of each modality and module on ReliaFusion-Net performance.

Ablation Settings	Accuracy	Precision	Recall	F1-score	Test AUC
Image only	0.7136	0.7768	0.7136	0.7307	0.7630
Temperature only	0.8505	0.8524	0.8405	0.8473	0.8353
Without bi-directional CoAttention	0.8636	0.8599	0.8636	0.8595	0.9147
Without FiLM	0.8864	0.9103	0.8864	0.8907	0.9692
Fixed weight fusion	0.9091	0.8939	0.8724	0.8823	0.9623
Feature concatenation	0.9091	0.9222	0.8464	0.8749	0.9653
**Image** **+** **Tabular data**	**0.9318**	**0.9346**	**0.9318**	**0.9326**	**0.9785**

Performance improves progressively with multimodal fusion and inclusion of Co-Attention and FiLM modules, with the full ReliaFusion-Net achieving the best results. FiLM, feature-wise linear modulation. Bold values indicate the best performance among the compared methods.

**Table 9:** Classification performance of ReliaFusion-Net on the test set with 95% confidence intervals.

Metrics	Value	95% CI
Sensitivity	0.9318	[0.7415, 0.9768]
Specificity	0.9375	[0.8500, 0.9754]
PPV	0.9346	[0.6647, 0.9685]
NPV	0.9677	[0.8898, 0.9911]
Accuracy	0.9318	[0.8591, 0.9684]

PPV, positive predictive value; NPV, negative predictive value; CI, confidence interval.

The five-fold cross-validation (CV) results of the ReliaFusion-Net with EdgeNext backbone (Table 10) further reinforce its robustness. The model achieved a mean accuracy of 0.8835 ± 0.0449, F1-score of 0.8722 ± 0.0468, and AUC of 0.9482 ± 0.0321, with narrow confidence intervals across all folds. The reported 95% confidence intervals are derived from five-fold subject-level cross-validation and reflect expected performance variability under repeated resampling, rather than uncertainty from a single held-out test split. The held-out test set was strictly reserved for one-time final evaluation to avoid information leakage. These findings demonstrate that the proposed ReliaFusion-Net model maintains generalization across folds and unseen subjects. Thermal eye imaging provides a non-invasive, contact-free method for binary classification of eyes with DR vs. healthy controls.

**Table 10:** Five-fold cross-validation performance of the ReliaFusion-Net EdgeNeXt backbone.

Metric	Mean	SD	95% CI (Low)	95% CI (High)	Folds
Accuracy	0.8835	0.0449	0.8485	0.9137	5
Precision	0.8814	0.0563	0.8457	0.9144	5
Recall	0.8655	0.0434	0.8276	0.8995	5
F1-Score	0.8722	0.0468	0.8347	0.9048	5
AUC	0.9482	0.0321	0.9053	0.9610	5

Five-fold cross-validation shows stable performance with low standard deviation, indicating good generalization of the proposed model. SD, standard deviation; CI, confidence interval; AUC, area under the curve.

Tables 8–10 summarize the cross-validation and hold-out performances, respectively. Although the model achieved higher performance on the held-out test set (Accuracy = 93.18%, AUC = 0.9785), the cross-validation estimates (Accuracy = 88.35 ± 4.49%, AUC = 0.9482 ± 0.0321) better reflect expected generalization behavior, as the test split composition influences results when the dataset is relatively small. The consistently high metrics with minimal deviation across both evaluations demonstrate that the proposed ReliaFusion-Net with the EdgeNeXt backbone achieves good stability and generalization without overfitting. To further examine model behavior, Table 11 presents classification performance at four operating points with varying decision thresholds. The high-sensitivity and F1-optimal points (threshold ≈ 0.69) achieved 91.67% sensitivity and 98.44% specificity, minimizing missed DR cases. The high-specificity point achieved the highest sensitivity (95.83%) with a slightly lower specificity (93.75%). The balanced point offers an equal trade-off between sensitivity and specificity. These results demonstrate that ReliaFusion-Net can be flexibly configured to suit different clinical priorities by adjusting the decision threshold.

**Table 11:** Operating points of ReliaFusion-Net at varying decision thresholds.

Point	Threshold	Sensitivity	Specificity	PPV	NPV	F1-score
High-sensitivity	0.6959	0.9167	0.9844	0.9565	0.9692	0.9362
High-specificity	0.4934	0.9583	0.9375	0.8519	0.9836	0.9020
F1-optimal	0.6875	0.9167	0.9844	0.9565	0.9692	0.9362
balanced	0.5489	0.9167	0.9375	0.8462	0.9677	0.8800

PPV, positive predictive value; NPV, negative predictive value; F1–F1-score. Thresholds were selected based on clinically relevant operating criteria.

The superior performance is primarily due to the efficient architectural design of EdgeNeXt, which combines lightweight convolutional processing with transformer-inspired contextual modeling. This enables effective extraction of both local and global features from thermal eye images with relatively low computational cost. Its strong feature representation capability also complements the co-attention mechanism, allowing improved interaction between modalities and more robust cross-modal fusion. Consequently, the framework can capture fine-grained thermal patterns more accurately and achieve better overall classification performance. These results align with prior studies reporting that EdgeNext variants offer an excellent accuracy–efficiency trade-off in medical imaging tasks (33, 34). The high AUC values and its lightweight architecture ensure deployability in real-time tele-ophthalmology environments.

### 3.4 Performance comparison with other backbone networks

The proposed ReliaFusion-Net was evaluated using multiple lightweight and efficient backbone networks, including mobileone (35), repvit (36), mobilenets (37), ghostnet (38), efficientnetb0 (39), and edgenext (34) to identify the optimal trade-off between classification performance and computational efficiency. As presented in Table 12, EdgeNeXt achieved the highest accuracy (93.18%), precision (93.46%), recall (93.18%), and F1-score (93.26%) with an AUC = 0.9785, confirming its superior discriminative capability for classification.

**Table 12:** Performance comparison of ReliaFusion-Net with different backbones.

Models	Accuracy	Precision	Recall	F1-score	Test AUC
**Mobileone**	0.6818	0.8531	0.6818	0.6959	0.9727
**Repvit**	0.7384	0.8077	0.7386	0.6383	0.5931
**mobilenets**	0.7500	0.7231	0.7500	0.7109	0.7799
**Ghostnet**	0.8409	0.8695	0.8409	0.8160	0.9486
**Efficientnetb0**	0.8977	0.8959	0.8977	0.8955	0.9707
**Edgenext**	**0.9318**	**0.9346**	**0.9318**	**0.9326**	**0.9785**

EdgeNeXt backbone achieves the best overall performance for thermal-based normal vs. eye with DR classification. AUC, area under the curve. Bold values indicate the best performance among the compared methods.

The computational characteristics summarized in Table 13 illustrate the balanced performance of EdgeNeXt, which offers moderate complexity (2.612 M parameters; 0.258 GFLOPs) while maintaining high classification accuracy. Indicating its potential suitability for deployment in resource-limited or real-time edge environments.

**Table 13:** Complexity and runtime metrics of backbones in the ReliaFusion-Net.

Models	Parameters_M	GFLOPs	Size_MB	Batchtime_ms	Per sample_ms
Mobileone	7.775	1.054	31.19	44.888	2.806
Repvit	6.617	0.848	25.9	21.056	1.316
Mobilenets	5.025	0.058	19.44	7.111	0.444
Ghostnet	9.139	0.188	35.38	707.559	44.222
Efficientnetb0	8.271	0.388	32.03	27.079	1.692
Edgenext	2.612	0.258	10.11	18.736	1.171

EdgeNeXt achieves the lowest parameter count and model size, indicating high efficiency with competitive computational cost. GFLOPs, giga floating point operations; MB, megabytes; ms, milliseconds.

EdgeNeXt was selected as the primary backbone because it achieved superior classification performance (accuracy = 93.18%, AUC = 0.9785) while maintaining balanced computational efficiency (2.612M parameters and 0.258 GFLOPs). Its robust generalization across thermal modalities makes it a reliable and computationally practical choice for real-time medical imaging applications.

The present work emphasizes model reliability and performance, evaluated using AUC, F1-score, precision, recall, and calibration consistency. The models with approximately ten million parameters or fewer were selected to ensure stable convergence, minimize overfitting, and maintain computational feasibility given the limited sample size. Among the tested architectures, EdgeNeXt achieved strong calibration, making it the most appropriate backbone for this study. An AUC of 0.9785 was obtained on the internal test set, reflecting perfect discrimination under current conditions.

In the present dataset, the age range considered for both normal and eye with DR subjects extends from 25 to 60 years. However, the distribution of samples across these age groups is not uniform, which is a common challenge in medical imaging datasets. Due to the progressive nature of eye with DR conditions, older individuals are more likely to belong to the diseased category, leading to greater class imbalance in the higher age groups. To account for this limitation and to further assess the robustness of the proposed framework, an age-stratified evaluation was carried out. The results of the age-stratified analysis, shown in Table 14, indicate that the proposed ReliaFusion-Net performs consistently well across all age groups. Among the subsets, the 40–50 age group yielded the best overall performance, achieving an accuracy of 94.44%, a F1-score of 0.9429, and an AUC of 0.9990. The 50–65 age group also showed high accuracy (92.59%) and AUC (0.9980); however, its comparatively lower F1-score (0.8125) and precision suggest the influence of stronger class imbalance within that subset. Overall, these findings demonstrate the strong age-wise generalization ability of the proposed model, while also highlighting the need for more balanced representation of older age groups in future data collection. However, further multicentre validation is needed to confirm generalizability. While latency, throughput, and model size were recorded, these factors were considered secondary to model performance in this phase. Future work will focus on reducing model complexity while sustaining high accuracy and AUC, supporting applications in telemedicine and portable screening systems.

**Table 14:** Age stratified analysis of the proposed ReliaFusion-Net.

Age Bin (years)	Samples	Normal	Diabetic	Accuracy	Precision	Recall	F1-score	AUC
25–40	32	20	12	0.9375	0.9000	0.9583	0.9227	0.9792
40–50	18	10	8	0.9444	0.9545	0.9375	0.9429	0.9990
50–65	34	4	30	0.9259	0.7500	0.9600	0.8125	0.9980

The model maintains consistently high performance across all age groups, with strong generalization in mid and older age bins. AUC, area under the curve.

## 4 Limitations and future work

The current study is limited by the relatively moderate dataset, which was collected from a single center and may influence the model's ability to generalize across broader populations. For effective clinical translation, future efforts should focus on expanding the dataset to include a more diverse, multicentre, and demographically balanced cohort. Although a significant age distribution difference was observed between the normal and diabetic groups, this reflects real-world screening populations. Future studies will incorporate age-matched comparison groups, including diabetic subjects without retinopathy, as well as longitudinal within-subject analysis to better isolate retinopathy-specific progression. Further research should aim to refine the framework to maintain classification accuracy while enhancing computational efficiency. Future work will also focus on developing a thermal-specific CNN for ocular thermal image classification. In this initial phase, left and right eye images were analyzed separately, and a small periocular region was included, consistent with prior literature. Improving screening precision will require region-of-interest (ROI)-based analysis targeting clinically significant ocular structures. The reliability mechanism was evaluated under clinically curated input conditions. Currently, the framework performs binary classification (Normal vs. eye with DR); subsequent work will extend this to a clinically stratified multi-stage classification (Normal, NPDR, PDR) to more accurately capture disease progression.

## 5 Conclusion

The proposed study introduces ReliaFusion-Net, a multimodal framework that combines thermal eye images and radiometric temperature features to support reliable classification of normal vs. eye with DR. The approach models cross-modal relationships through bidirectional co-attention and feature-wise modulation, while a reliability-gating mechanism adaptively balances the contribution of each modality based on confidence levels. Experiments conducted on a clinically curated dataset of 558 thermal eye images demonstrated consistent performance, with ReliaFusion-Net achieving 93.18% accuracy, and 93.26% F1-score, along with strong cross-validation results (Accuracy = 88.35 ± 4.49%, AUC = 0.9482 ± 0.0321). In addition to high performance, the framework showed resilience to variations in data quality, addressing a major challenge in multimodal medical image analysis. By integrating temperature-derived physiological indicators with image features, ReliaFusion-Net facilitates non-invasive discrimination between normal eyes and those with diabetic retinopathy. The use of low-cost thermal imaging and lightweight deep architectures suggests potential applicability in resource-constrained environments, though broader suitability for tele-ophthalmology and point-of-care screening will require validation through prospective, multi-center studies. Future work will focus on expanding the dataset, extending classification to multi-stage DR severity grading, and optimizing the architecture for real-time deployment on portable screening platforms.

## Data Availability

The raw data supporting the conclusions of this article will be made available by the authors, without undue reservation.
